# Development of 10 single‐copy nuclear DNA markers for *Euchresta horsfieldii* (Fabaceae), a rare medicinal plant

**DOI:** 10.1002/aps3.1178

**Published:** 2018-09-21

**Authors:** Arief Priyadi, Chao Feng, Ming Kang, Hongwen Huang

**Affiliations:** ^1^ Bali Botanical Garden Indonesian Institute of Sciences (LIPI) Tabanan 82191 Bali Indonesia; ^2^ Key Laboratory of Plant Resources Conservation and Sustainable Utilization South China Botanical Garden Chinese Academy of Sciences Guangzhou 510650 People's Republic of China; ^3^ University of Chinese Academy of Sciences Beijing 100049 People's Republic of China

**Keywords:** *Euchresta horsfieldii*, Fabaceae, genetic diversity, RNA‐Seq, single‐copy nuclear DNA (scnDNA) markers

## Abstract

**Premise of the Study:**

*Euchresta horsfieldii* (Fabaceae) is a rare and endangered medicinal plant in Indonesia with restricted distribution. Single‐copy nuclear DNA (scnDNA) markers were developed for this species to facilitate further investigation of genetic diversity and population structure.

**Methods and Results:**

We performed RNA‐Seq and de novo assembly of the transcriptome. Ten primer sets were developed for *E. horsfieldii*, all of which also amplified in *E. japonica* and *E. tubulosa*.

**Conclusions:**

These scnDNA markers will be an important resource for the study of genetic diversity and population structure of *E. horsfieldii* and other species in the genus *Euchresta*.

*Euchresta horsfieldii* (Lesch.) Benn. is a perennial shrub distributed in Bhutan, China, India, Indonesia, Laos, Nepal, the Philippines, Thailand, and Vietnam (Sun and Larsen, 2010). In Indonesia, the natural habitats of *E*. *horsfieldii* are restricted to the middle range of rainforests at 1300–2400 m above sea level in Sumatra, Java, and Bali (van Steenis, 2006). *Euchresta horsfieldii* is used in traditional Indonesian herbal medicine, and its pharmacological properties have been evaluated for antitumor, anti‐oxidant, and lipid‐reducing agents (Li et al., 2014). Despite its potency as an Asian medicinal plant, its rarity, and its endangered status, studies of the conservation genetics of the species are still scarce. Hama et al. (2009) developed a set of microsatellite primers for *E. japonica* Hook. f. ex Regel and performed cross‐amplification in *E. formosana* (Hayata) Ohwi. We tested these markers in *E. horsfieldii*, and only two out of nine primers amplified. Thus, we developed a new set of single‐copy nuclear DNA (scnDNA) markers for *E. horsfieldii* to allow further study of its population genetic diversity and structure. We used RNA‐Seq, transcriptome de novo assembly, and subsequent bioinformatics workflows to develop scnDNA markers. Ten primer pairs of scnDNA markers were obtained for *E. horsfieldii* and *E. japonica*. We successfully used these primers to assess polymorphism of three wild populations of *E. horsfieldii* from Indonesia and one population of *E. tubulosa* Dunn from China. Therefore, this set of scnDNA markers should be helpful in population genetic studies of *E. horsfieldii* and related taxa.

## Methods and results

A stem cutting of *E. horsfieldii* was collected from Tapak Hill, Bali, Indonesia, and cultivated in the greenhouse of South China Botanical Garden, Guangdong, China. Fresh leaves were harvested from the 3‐month‐old plants grown from this cutting for RNA extraction. Library construction of cDNA and the transcriptome sequencing followed protocols by Ai et al. (2015), with some modifications. Total RNA was isolated from approximately 100 mg of fresh leaf tissue of *E. horsfieldii* with the TransZol Up Plus RNA Kit (TransGen Biotech Co., Beijing, China), following the manufacturer's protocols. Total mRNA was isolated using Oligo(dT) cellulose, and subsequently first‐ and second‐strand cDNA were synthesized. Sequencing adapters were ligated to short fragments. Agarose gel electrophoresis was used to separate fragments with lengths of approximately 350 bp, which were then selected as sequencing templates for PCR amplification. Transcriptome sequencing was performed on an Illumina HiSeq X Ten System (Illumina, San Diego, California, USA) that generated 150‐bp paired‐end raw reads. The library construction and sequencing was carried out by a commercial company (Novogene Bioinformatics Institute, Beijing, China).

Raw reads were filtered by removing the low‐quality sequences using QC_pe pipeline (Feng et al., 2017), and clean data were used for de novo assembly using Trinity (Grabherr et al., 2011). Transcript quantification was performed using the software package RNA‐Seq by Expectation Maximization (RSEM; Li and Dewey, 2011), and only assembled genes with fragments per kilobase of transcript per million mapped reads (FPKM) values greater than 1 were selected for subsequent analysis. Coding regions within these unigenes were predicted by TransDecoder version 5.0.1 (https://github.com/TransDecoder). We performed Pfam and BLASTP searches of these protein‐coding genes against UniProtKB/Swiss‐Prot to predict their putative functions. Their ortholog groups were compared against *Ricinus communis* L., *Arabidopsis thaliana* (L.) Heynh., *Oryza sativa* L., and *Physcomitrella patens* (Hedw.) Bruch & Schimp. and identified using an online version of OrthoMCL‐DB (Chen et al., 2006; http://orthomcl.org/orthomcl/). These ortholog groups were treated as putative single‐copy genes (scnDNA). Approximately 21 million Illumina paired‐end clean reads were generated (National Center for Biotechnology Information [NCBI] Sequence Read Archive [SRA] accession no. SRP149026). Clean reads were assembled into 61,796 unigenes with an N50 length of 2160 bp. Among these, 49,804 unigenes with FPKM greater than 1 were obtained, 27,405 protein‐coding genes were predicted, and 1017 putative scnDNA were identified. We randomly selected 24 of these putative scnDNA for initial design of 72 PCR primers using Primer‐BLAST (Ye et al., 2012).

To validate the scnDNA markers, genomic DNA was extracted from two individuals each of *E. horsfieldii* (population BLBG) and *E. japonica* (populations SCB1, SCB2) (Appendix 1). Validation was done separately in these two species. DNA was extracted from approximately 15–20 mg of silica gel–dried leaf samples using the Plant Genomic DNA extraction kit (BioTeke, Beijing, China), following the manufacturer's instructions. DNA amplification was performed in a 20‐μL reaction mixture containing 10 μL of 2× EasyTaq PCR SuperMix (TransGen Biotech Co.), 0.5 μL each of forward and reverse primer, 8.5 μL of ddH_2_O, and approximately 50 ng of template DNA. The PCR program was set as one cycle of 5 min at 95°C; 35 cycles of 30 s at 94°C, 90 s at 55°C, 60 s at 72°C; and a final extension of 10 min at 72°C. For amplicon quality and quantity check, each PCR product was run for 15 min of electrophoresis in 1% agarose gel at 120 V. Amplicons with only one clear band were sequenced using an ABI 3730xl DNA Sequencer (Tsingke Biological Technology, Guangzhou, China). Ten primer pairs showed single clear bands and good electropherogram quality from Sanger sequencing. Sequence data were then read, trimmed, and exported to FASTA in Chromas version 2.6.2 (Technelysium, South Brisbane, Queensland, Australia; http://technelysium.com.au). FASTA sequences were aligned using the MUSCLE algorithm available in MEGA 7.0 (Kumar et al., 2016) and then formatted manually to PHYLIP file format as input for further analysis.

These 10 primers were assessed for polymorphism in three populations of *E. horsfieldii* from Indonesia and one population of *E. tubulosa* from China, following the same protocol described above for marker validation. In total, we collected 38 wild individuals of *E. horsfieldii* from Indonesia and six individuals of *E. tubulosa* from China. Voucher specimens of *E. horsfieldii* were deposited in the Herbarium Hortus Botanicus Baliense (THBB), Bali Botanic Garden, Indonesian Institute of Sciences (LIPI), Bali, Indonesia (Appendix 1). PCR primer pairs and characteristics of the 10 newly developed scnDNA markers, GenBank accessions, and BLASTN hits are presented in Table 1. Genetic diversity measures of all samples derived from pairwise number of site differences, including nucleotide diversity (π), Watterson estimator (*θ*_w_), and related measures, were calculated using DnaSP version 5.10 (Librado and Rozas, 2009) (Table 2). The average number of alleles was 5.9 (5 to 7), π was 5.03 × 10^−3^ (1.75 × 10^−3^ to 7.5 × 10^−3^), and *θ*_w_ was 4.01 × 10^−3^ (1.65 × 10^−3^ to 7.23 × 10^−3^). There was no significant Tajima's D; however, EhoScn04a and EhoScn16a showed significant negative Fay and Wu's H, indicating non‐neutrality of these two loci. Most loci showed no linkage disequilibrium after 10,000 permutations in Arlequin version 3.5.2.2 (Excoffier et al., 2005), except EhoScg15b and EhoScg24b in population SCHU. For the population genetic analysis, the PHYLIP file format was first converted to STRUCTURE input using SPADS 1.0 (Dellicour and Mardulyn, 2014), followed by conversion to GENEPOP format using PGDSpider2 (Lischer and Excoffier, 2012). Allelic richness of each locus and population and number of private alleles (Appendix 2) were generated by PopGenReport version 3.0.0 (Adamack and Gruber, 2014). The average allelic richness was 1.25 (1 to 2.56) and average number of private alleles was 10.2 (3 to 21). These figures show that the genetic diversity of *Euchresta* spp. vary across species. Within each population, *E. horsfieldii* are homozygotes and show no genetic variation, but variations are detected among populations. The KTCN and MRPI populations were found to share seven common alleles and to each have three private alleles, whereas all 10 alleles from the BALI population are private.

**Table 1:** PCR primers and characteristics of 10 scnDNA markers developed for *Euchresta horsfieldii*

Locus	Primer sequences (5′–3′)	*T*_a_ (°C)	Fragment size (bp)	BLASTN top hit description	*E*‐value	Closest species	GenBank accession no.
EhoScn01a	F: AAGTTCCGCTTCCTCGAATC	52.5	646	AdoMet‐dependent rRNA methyltransferase SPB1	2e‐99	*Cajanus cajan*	MH248777–MH248783
	R: GTAATTACCTTCGCCTGGGG						
EhoScn02b	F: GCGAAAAACCTCGTACTTGC	51.5	671	Diphthamide biosynthesis protein 1	0	*Lupinus angustifolius*	MH269247–MH269251
	R: CTCTGCAGCTACACTCACAG						
EhoScn04a	F: ATGGACACCCACTCTTCTCA	51.5	529	Histone acetyltransferase GCN5	1e‐116	*Glycine max*	MH269252–MH269256
	R: ACTCTCTTCTCTGGCAGTGT						
EhoScn15b	F: TCTACTTCCGACCCCTTTGT	51.8	591	Protein ABCI7, chloroplastic	9e‐103	*Lupinus angustifolius* cv. Tanjil	MH269257–MH269263
	R: CTCCAATGGCAACCTCCAAT						
EhoScn16a	F: AAGCAATGCCGAGACGAGAA	53.5	445	DNA/RNA‐binding protein KIN17	0	*Glycine max*	MH269264–MH269268
	R: CCTCGGACACTCAACCTGTG						
EhoScn17a	F: ATCGGCACTCTTCTGAGACTGA	53.0	538	F‐box/LRR‐repeat protein 10	0	*Lupinus angustifolius* cv. Tanjil	MH269269–MH269274
	R: ACCTCATCATAATCACGCGCA						
EhoScn20a	F: CCAGGTTAAGGGTCGCAAGT	54.0	553	Pentatricopeptide repeat‐containing protein At3g16010	0	*Lupinus angustifolius* cv. Tanjil	MH269275–MH269280
	R: GCCTCAGAAGGTGGAGCTTT						
EhoScn21c	F: ATCACAACCAGCCCAACCAA	53.6	709	Mediator of RNA polymerase II transcription subunit 4	0	*Lupinus angustifolius* cv. Tanjil	MH269281–MH269285
	R: TGGATTGGCATCCAAAGGCT						
EhoScn23c	F: CCACTTCGCCACCAAGTACA	54.0	439	Pentatricopeptide repeat‐containing protein At1g51965, mitochondrial	2e‐108	*Lupinus angustifolius* cv. Tanjil	MH269286–MH269291
	R: TCTAAATCCTGGCCGTTCCC						
EhoScn24b	F: AAGAACCTTCGTTATCCACAGCA	52.6	631	Pentatricopeptide repeat‐containing protein At2g01860	0	*Lupinus angustifolius* cv. Tanjil	MH269292–MH269298
	R: TAATAAGGGCACCACATGCCT						

Note

*T*_a_ = annealing temperature.

**Table 2:** Genetic properties of the 10 newly developed scnDNA markers of *Euchresta horsfieldii*, cross‐amplified in *E. japonica* and *E. tubulosa*

Locus	*n*	Length	*S*	*p*_n_	*A*	*k*	*h*	π (× 10^−3^)	*θ*_W_ (× 10^−3^)	Tajima's D	Fay & Wu's H
EhoScn01a	96	646	24	0.0372	7	4.848	0.752	7.50	7.23	0.1116	0.3725
EhoScn02b	96	671	10	0.0149	5	3.403	0.629	5.07	2.9	1.9226	−1.4166
EhoScn04a	96	529	9	0.0170	5	2.547	0.751	4. 87	3.72	0.7930	−6.6328^a^
EhoScn15b	96	591	16	0.0271	7	3.494	0.635	5.91	5.27	0.3425	0.2169
EhoScn16a	96	445	7	0.0157	5	1.479	0.629	3.32	3.5	−0.1232	−3.7282^a^
EhoScn17a	96	538	8	0.0149	6	2. 377	0. 627	4.42	2.90	1.2839	−1.6326
EhoScn20a	96	553	13	0.0235	6	3.729	0.752	6.74	4.58	1.2838	0.9755
EhoScn21c	96	709	6	0.0085	5	1.238	0. 629	1.75	1.65	0.1347	0.4301
EhoScn23c	96	439	8	0.0182	6	1.857	0. 629	4.23	3.55	0.4701	0.2507
EhoScn24b	96	631	15	0.02378	7	3.854	0.631	6.32	4.79	0.8896	−0.9234

Note

*A* = number of alleles; *h* = haplotype diversity; *k* = average number of nucleotide differences; *n* = number of sequences used (consists of four sequences of *E. japonica*, 12 sequences of *E. tubulosa*, and 80 sequences of *E. horsfieldii*); *p*_n_ = proportion of polymorphic sites; π = nucleotide diversity; *θ*_w_ = Watterson estimator per site from *S*;* S* = variable sites.

a Significant (α = 0.01).

## Conclusions

In this study, we demonstrated the application of RNA‐Seq to develop scnDNA markers for *E. horsfieldii*, and these markers were cross‐amplified to *E. japonica* and *E. tubulosa*. These scnDNA markers will provide useful resources to study the population genetic diversity and population structure of these rare medicinal plants.

## Author contributions

M.K. conceived and designed the project. A.P. carried out the field and laboratory works supervised by H.H. C.F. conducted bioinformatics analyses. A.F. and C.F. wrote the manuscript. All authors read and approved the final version of the manuscript.

## Data accessibility

Illumina paired‐end clean reads were deposited to the National Center for Biotechnology Information (NCBI) Sequence Read Archive (SRA; accession no. SRP149026). Sequence information for the developed primers has been deposited to NCBI; GenBank accession numbers are provided in Table 1.

## Appendix 1 Voucher information for *Euchresta* species used in this study.

**Table:**

Species	Voucher specimen accession no.	Collection locality	Geographic coordinates	Population code	No. of individuals genotyped
*E. horsfieldii* (Lesch.) Benn.	E19910118^c^	Bali, Indonesia (living collection of Bali Botanic Garden)	8.28370S, 115.13702E (8.27681S, 115.15338E)	BLBG	2
	AP231^a^	Bedugul, Tabanan, Bali, Indonesia	8.28370S, 115.13702E	BALI	14
	AP233^a^	Mt. Marapi, Kotobaru, West Sumatra, Indonesia	0.39433S, 100.42735E	MRPI	14
	AP234^a^	Mt. Kerinci, Kutacane, Aceh, Indonesia	3.35859N, 97.70061E	KTCN	10
*E. japonica* Regel	20060969/N27‐0002^c^	Lechang, Guangdong, China (living collection of SCBG)	— (23.18033N, 113.35351E)	SCB1	1
	20100407/11‐0004^c^	Sangzhi, Hunan, China (living collection of SCBG)	29.11456N, 110.46506E (23.18033N, 113.35351E)	SCB2	1
*E. tubulosa* Dunn	PE 00305297^b^	Mt. Emei, Sichuan, China	29.55253N, 103.34494E	SCHU	6

Note

AP = Arief Priyadi, collector.

a Vouchers deposited at the Herbarium Hortus Botanicus Baliense (THBB), Bali Botanic Garden, Indonesian Institute of Sciences (LIPI), Bali, Indonesia.

b No vouchers collected during this study. Voucher information is from the Herbarium of the Institute of Botany (PE), Chinese Academy of Sciences; accessed from the Chinese Virtual Herbarium (http://www.cvh.ac.cn).

c Living collections of the South China Botanical Garden (SCBG), Guangzhou, Guangdong, China, or Bali Botanic Garden, LIPI, Tabanan, Bali, Indonesia.

## Appendix 2 Allelic richness and private alleles assessed at the population level using 10 scnDNA markers developed for *Euchresta horsfieldii*.

**Table:**

Population (Species)	*N*	Allelic richness per locus per population										No. of private alleles
		01a	02b	04a	15b	16a	17a	20a	21c	23c	24b	
SCB1, SCB2 (*E. japonica*)	2	2.557	1.971	1	1.971	1.971	2.557	1.971	1.786	2.557	1.971	21
SCHU (*E. tubulosa*)	6	1	1	1	1.854	1	1.312	1	1	1	2.171	14
KTCN (*E. horsfieldii*)	10	1	1	1	1	1	1	1	1	1	1	3
MRPI (*E. horsfieldii*)	14	1	1	1	1	1	1	1	1	1	1	3
BALI, BLBG (*E. horsfieldii*)	16	1	1	1	1	1	1	1	1	1	1	10

Note

*N* = number of individuals sampled.
