# Corrigendum

**DOI:** 10.1002/aps3.1252

**Published:** 2019-06-07

In our paper, “Development of 10 single‐copy nuclear DNA markers for *Euchresta horsfieldii* (Fabaceae), a rare medicinal plant,” the author affiliations were listed in the incorrect order. They have been updated to reflect:

Arief Priyadi^1,2,3^, Chao Feng^1^, Ming Kang^1^, and Hongwen Huang^1,4^

^1^Key Laboratory of Plant Resources Conservation and Sustainable Utilization, South China Botanical Garden, Chinese Academy of Sciences, Guangzhou 510650, People's Republic of China

^2^University of Chinese Academy of Sciences, Beijing 100049, People's Republic of China

^3^Bali Botanical Garden, Indonesian Institute of Sciences (LIPI), Tabanan 82191, Bali, Indonesia

^4^Author for correspondence: huanghw@mail.scbg.ac.cn

We apologize for this error.
